# Evaluating the Feasibility of a Software Prototype Supporting the Management of Multimorbid Seniors: Mixed Methods Study in General Practices

**DOI:** 10.2196/12695

**Published:** 2019-07-04

**Authors:** Christine Kersting, Birgitta Weltermann

**Affiliations:** 1 Institute for General Medicine University Hospital Essen University of Duisburg-Essen Essen Germany; 2 Institute of General Practice and Family Medicine University Hospital Bonn University of Bonn Bonn Germany

**Keywords:** patient care management, primary health care, clinical decision support systems, electronic health record, reminder system, health information technology

## Abstract

**Background:**

Longitudinal, patient-centered care represents a challenge for general practices. Decision support and reminder systems can offer targeted support.

**Objective:**

The objective of this study was to follow a user-oriented, stepwise approach to develop an add-on for German electronic health record (EHR) systems, which aims to support longitudinal care management of multimorbid seniors, using a flag system displaying patient-centered information relevant for comprehensive health care management. This study evaluated the prototype’s feasibility from both a technical and users’ perspective.

**Methods:**

The study was conducted with 18 general practitioners (GPs) and practice assistants (PAs) from 9 general practices using a mixed methods approach. In all practices, 1 GP and 1 PA tested the software each for 4 multimorbid seniors selected from the practice patient data. Technical feasibility was evaluated by documenting all technical problems. To evaluate the feasibility from the users’ perspective, participants’ responses during the software test were documented. In addition, they completed a self-administered questionnaire, including the validated System Usability Scale (SUS). Data were merged by transforming qualitative data into quantitative data. Analyses were performed using univariate statistics in IBM SPSS statistics.

**Results:**

From a technical perspective, the new software was easy to install and worked without problems. Difficulties during the installation occurred in practices lacking a 64-bit system or a current version of Microsoft .NET. As EHRs used in German practices do not provide an interface to extract the data needed, additional software was required. Incomplete flags for some laboratory data occurred, although this function was implemented in our software as shown in previous tests. From the users’ perspective, the new add-on provided a better overview of relevant patient information, reminded more comprehensively about upcoming examinations, and better supported guideline-based care when compared with their individual practice strategies. A total of 14 out of 18 participants (78%) were interested in using the software long-term. Furthermore, 8 of 9 GPs were willing to pay 5 to 25 Euros (mean 14.75, SD 5.93) monthly for its use. The usability was rated as 75% (43%-95%).

**Conclusions:**

The new EHR add-on was well accepted and achieved a good usability rating measured by the validated SUS. In perspective, the legally consolidated, standardized interface to German EHRs will facilitate the technical integration. In view of the high feasibility, we plan to study the software’s effectiveness in everyday primary care.

**Trial Registration:**

German Clinical Trials Register DRKS00008777; https://www.drks.de/drks_web/navigate.do? navigationId=trial.HTML&TRIAL_ID=DRKS00008777

## Introduction

### Background

Health care management of seniors (persons aged ≥65 years) is complex as more than 55% are multimorbid [1]. Electronic health records (EHRs) have the potential to support care management, but were shown to insufficiently support longitudinal patient-centered management as they (1) require high user-system interaction and work slow, (2) lack user-friendliness and orientation to typical health care processes, (3) provide insufficient interoperability, (4) offer insufficient service if help is needed, and (5) inadequately distinguish between information relevant and irrelevant for patient care [2-7]. Surveys evaluating the use and functional capacity of EHR in German primary care revealed a high implementation rate of EHR systems but little multifunctional capacity, especially with regard to reminder and recall systems [2,6,8,9].

Considering that the demographic change will lead to an increasing number of seniors and complex, multimorbid patients, health information technologies (IT), which adequately support health care management, are needed. EHR add-ons such as clinical decision support systems (CDSS), including reminder systems, can provide targeted support as they were shown to effectively support management of, for example, diabetes or hypertension [10-14]. Limiting the benefit of such disease-specific CDSS, a survey among primary care physicians revealed that physicians are more willing to accept new IT solutions, when supporting the management of elderly patients with multiple conditions or polypharmacy [15]. In addition, many systems do not adequately meet physicians’ needs, even though an early involvement of the target group into software development is described as key facilitator for software acceptance [16-18] and is therefore highly recommended [19].

### Objectives

**Figure 1:**
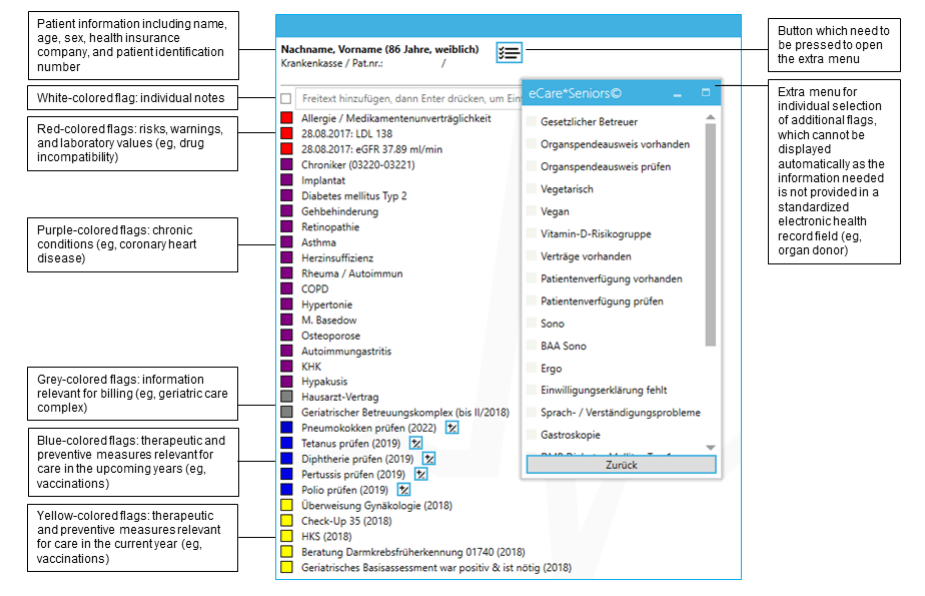
Example of the patient-centered flag system of eCare*Seniors displaying information relevant to health care management. Note: As the software was developed for German general practitioners’ practices, the screenshot is in German, but includes an English explanation.

To overcome deficits of the current EHR solutions, we developed a new software add-on for German EHR systems. The add-on named eCare*Seniors aims to provide a quick and intuitive overview of each patient’s care needs to adequately support comprehensive and longitudinal patient-centered care management not only for multimorbid seniors in general practices, but for other patient groups as well [20,21]. To provide a quick overview of each patient’s care needs, relevant information is extracted from a patient’s EHR and reedited using so-called flags, which are a combination of colored fields and short keywords (Figure 1) [20,21]. The theoretical concept is based on the chronic care model [22-24], whereas from a functional perspective, eCare*Seniors is classified as a CDSS, integrating the functions of a reminder, advisor, and critic [25-27]. Further details on the new add-on and its functions are published elsewhere [20].

Following the recommendation for the development of health IT [19], eCare*Seniors was developed using a user-oriented, stepwise approach. First, an assessment of the status quo showed that German general practitioners (GPs) and practice assistants (PAs) are not satisfied with current EHR solutions and had started to self-design reminder systems within the EHR to maintain an overview of each patient’s care [5,28]. In a second step, we presented the concept for the new software add-on to GPs and PAs who generally welcomed the approach but expressed a desire for configuration options to adapt the software to their individual practice needs. On the basis of this target group information, we then implemented the software prototype in a third step. In this paper, we present the results of the feasibility study of the new software add-on, which was the fourth step of the development: the software was tested in GP practices to assess the feasibility from both a technical and users’ perspective.

## Methods

### Study Design

The feasibility study was conducted using a simultaneous mixed methods design involving qualitative and quantitative approaches [29]. According to recommendations on how to design feasibility studies, we focused on the 5 key criteria, integration, implementation, acceptability, demand, and practicability [30], which we subdivided into 2 categories:

1. Technical feasibility: Integration of the new add-on into existing IT infrastructure and implementation of the new add-on with regard to its success or failure of execution during the work process.
2. Feasibility from the users’ perspective: Demand for and acceptance of the new add-on within the target group, and assessment of the usability from the users’ perspective. Usability as one of the most important indicators for feasibility was measured using the validated System Usability Scale (SUS) [31,32].

### Setting and Recruitment

As recommendations on how to perform usability studies recommend sample sizes of 3 to 20 participants and the SUS was even shown to provide consistent results in study groups of 12 persons [33,34], we targeted a sample size ≥12 participants. The study was performed in a convenience sample of GP practices of the practice networks of the Institute for General Medicine, University of Duisburg-Essen, Germany, and the Institute for Family Medicine and General Practice of the University of Bonn, Germany (both >100 practices), with 1 GP and 1 PA each. Owing to the lack of a standardized, open interface of existing EHRs, a second software was used to extract the data needed for our new software add-on. To assure this connectivity to existing EHRs, only practices that apply one of 13 specific EHR solutions, which are used by about 85% of all German GP practices [35], were eligible to participate. As the new software is meant to be usable and feasible for every practice, no further inclusion and exclusion criteria were defined. Considering a participation rate of at least 40%, 30 participants from 15 practices who met the inclusion criteria and were located in the Greater Essen region, Germany, or those who already participated in an earlier step of the study were contacted and invited to participate.

### Data Collection and Data Management

All participating GP practices were visited twice by a research team member of the institute. During the first visit, the researcher installed the prototype of the newly developed add-on eCare*Seniors. During the second visit, the new add-on was introduced to the practice and tested by 1 GP and 1 PA per practice with patient data. To evaluate the technical feasibility, all problems that occurred during the installation process and when executing the new add-on during the test were documented. To evaluate the feasibility from the users’ perspective, data were collected using a prestructured interview guide for documentation of the software test and a self-administered questionnaire, which was completed by each participant after the software test:

(1) Documentation of the software test: First, the GP was asked to configure the software according to the practice’s needs. The preferences were documented using a checklist. Afterward, the software was tested independently by the GP and the PA. Both accessed 4 complex patients (defined as seniors aged 65 years or above with 2 or more chronic conditions) within the prototype of the new add-on and within the practice’s EHR and compared both systems based on the following questions:

- Is there any information on this patient you would have forgotten had you not used the new add-on? (yes/no)
- Does the new add-on display any information at first glance which you do not see instantly in the patient’s EHR? (yes/no)
  - What information? (open answer)
  - Is this information important? (yes/no)
- Is there any information available at first glance in the patient’s EHR which you do not see instantly in the new add-on? (yes/no)
  - What information? (open answer)
  - Is this information important? (yes/no)

To complete the test, GPs and PAs clicked through the remaining menu of the software add-on and applied the method of thinking aloud to help the researcher identify problems regarding comprehensibility and handling.

(2) Written questionnaire including the following aspects:

- German version of the validated SUS to evaluate the usability of software solutions based on 10 questions on a 5-point Likert scale [31,32]
- Two open-ended questions to request which aspects of the software were liked most and which aspects needed to be optimized
- One close-ended question to assess whether participants would choose to use the new add-on long-term
- Five questions comparing the new software add-on with the current practice-specific care management strategies to assess the aspects of quick overview, reminder functionality, support of individual, guideline-oriented care planning, support of time-efficient processes, and financial benefits on a 5-point Likert scale
- One open-ended question to assess how much money (in Euros) the potential users would be willing to pay for such software per month.

In addition, sociodemographic characteristics of the participants were assessed.

All data were entered manually in an access-restricted database in IBM SPSS Statistics for Windows, version 24 (IBM Corp).

### Data Analysis

Following an approach of Creswell and Plano Clark for integrating quantitative and qualitative data during the analysis process, we merged the data by transforming qualitative into quantitative data using categorization [29]. After that, all data were analyzed using descriptive statistics in SPSS. The usability was analyzed by determining the mean SUS score. The score can assume values between 0 and 40. Results were interpreted in percentage, that is, each score was multiplied by the factor 2.5 [31,32]. A SUS score ≥70% denotes good usability, whereas a score ≤50% indicates a considerable need for improvement [32].

### Ethical Considerations

Ethical approval was obtained from the Ethics Committee of the Medical Faculty of the University of Duisburg-Essen (reference number: 14-5980-BO; date of approval: June 1, 2015). All participants received written information and signed the informed consent forms, which are stored at the institute. All members of the practice network had provided written informed consent for the pseudonymized analysis of data on practice characteristics within the scope of scientific research of the institute.

## Results

### Study Characteristics

Of the 30 participants from 15 practices invited, 6 participants from 3 practices refused to participate, yielding an initial response rate of 80% (24/30). As detailed below, 3 practices with 6 participants willing to participate used an operating system, which was not supported by the new software (32-bit instead of the required 64-bit). Finally, 18 participants (9 GPs, 9 PAs) from 9 practices took part. The practices used 4 different EHR software solutions covering about 26% of all EHR solutions installed in German GP practices [35]. All practices participated with 1 GP and 1 PA each (N=18). Furthermore, 5 of these practices were group practices. The GPs were aged between 40 and 56 years (mean 47.4, SD 5.2); 8 of them were male. All PAs were female and aged between 22 and 60 years (mean 36.7, SD 14.3). All participants tested the software add-on with 4 patient charts, yielding a total of 72 patient tests.

### Technical Feasibility

The following technical problems were observed during the installation processes:

- As mentioned above, the new software could not be installed in 3 practices as they used a 32-bit operating system rather than the required 64-bit system.
- Installation was delayed in 4 practices as their Microsoft .NET program, which is required to run the desktop application of our software, needed to be updated.
- One of the practices did not have sufficient administration rights to install the software. To continue the installation process, access data had to be requested from the responsible software publisher.

Once these installation problems were resolved, the software prototype worked without problems. No technical problems occurred when executing the software during the practice tests. Nevertheless, in all 9 practices, few flags were not displayed in eCare*Seniors. In all 9 practices, the present year was named as due date in all flags on upcoming vaccinations. Compared with the information available within the patient charts, flags on chronic medication were incomplete in 4 practices, those on chronic diseases in 3 practices, and those on laboratory values in 1 practice. As all these flags were completely and successfully implemented in tests conducted during the software development, these problems are likely because of connectivity problems of the second software used to extract EHR data.

### Feasibility From the Users’ Perspective

During the practice test, each participant compared the information displayed at first glance in the practice’s EHR with the flags presented by eCare*Seniors. For 69 of the 72 (96%) patient charts selected for the test, participants stated that information which they consider important for comprehensive care management was available at first glance only in eCare*Seniors but not in their EHR (Table 1).

During the interviews alongside the chart tests, for 54 of the 72 (75%) patient cases tested, participants named some additional information which they would like to be presented in eCare*Seniors. The researchers judged the following information as reasonable to be evaluated for integration into a future version of eCare*Seniors:

- More detailed information on chronic diseases, for example, renal impairment in diabetic patients (mentioned in 14 of the 54 patient charts, 26%)
- More space for freely formulated notes and risks (12/54, 22%)
- More detailed information on allergies (11/54, 20%)
- More detailed information on chronic medication (9/54, 17%)
- Residential care/home visit patient (8/54, 15%)
- More detailed information on physical parameters such as height and weight (5/54, 9%)

Other wishes mentioned need be evaluated with regard to technical implementability (eg, the availability of an electronic medication plan or the date of the next disease management program examination).

**Figure 2:**
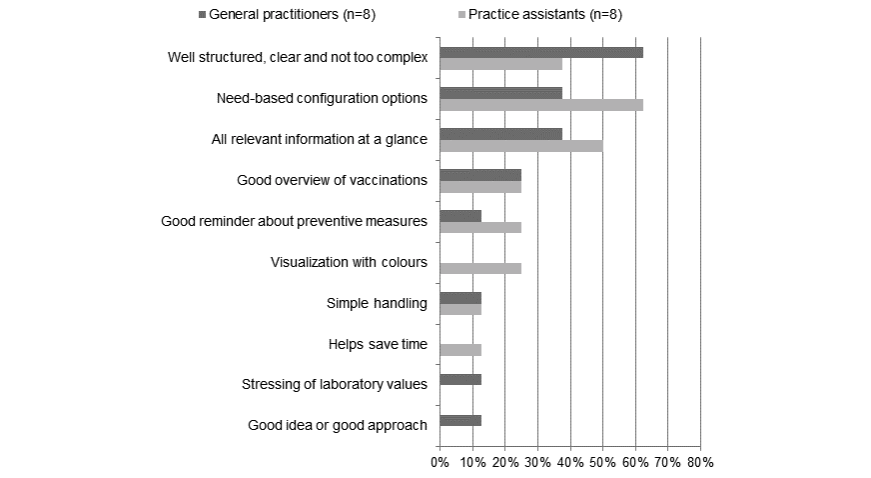
Positive aspects of eCare*Seniors according to general practitioners and practice assistants.

**Figure 3:**
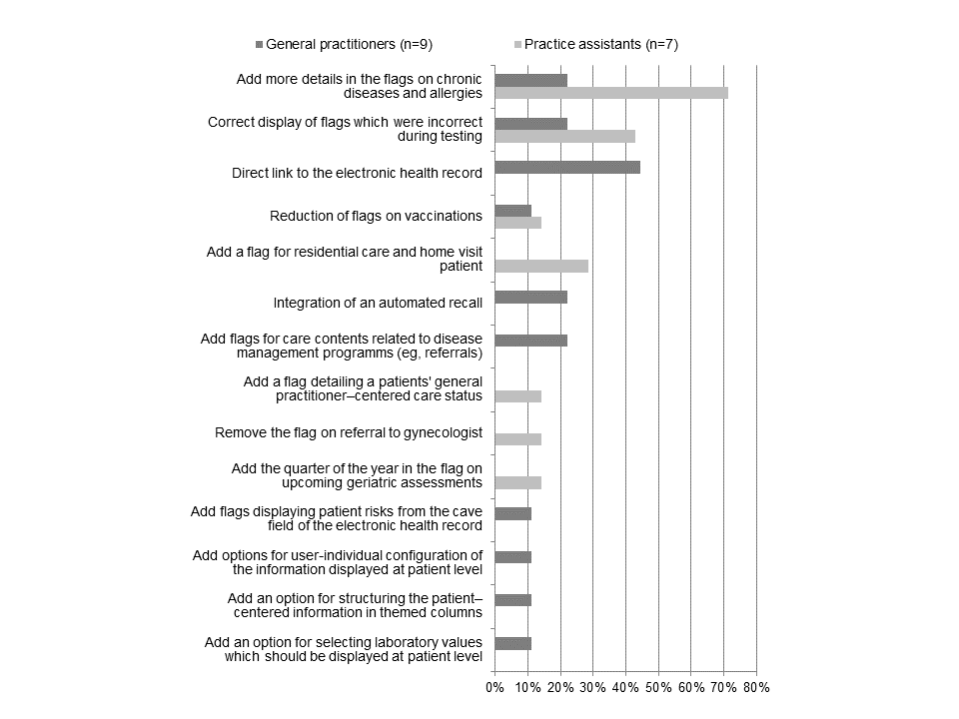
Optimization requirements of eCare*Seniors according to general practitioners and practice assistants.

Furthermore, 8 GPs and 8 PAs each (16/18, 89%) specified one or more aspect of the new software they liked most (Figure 2) and 9 GPs and 7 PAs (16/18, 89%) specified one or more aspect which needed to be optimized (Figure 3).

**Table 1:** Practice test: information only available at first glance in the new software and its relevance for care management.

Information displayed in eCare*Seniors	Information was not available in patients’ electronic health records at first glance (N=69), n (%)	Information was judged relevant for care management of this patient	
		n (%)	N
Upcoming vaccinations	64 (93)	60 (94)	64
Upcoming cancer screening including referrals to specialists	33 (48)	28 (85)	33
Laboratory findings	12 (17)	12 (100)	12
Chronic diseases (eg, rheumatoid arthritis)	8 (12)	8 (100)	8
Warnings/allergies	4 (6)	4 (100)	4
Billing codes	3 (4)	3 (100)	3
Geriatric care complex	2 (3)	2 (100)	2
Participation in a disease management program	1 (1)	1 (100)	1

The results of the questionnaire survey showed that 14 of the 18 participants (78%; 7 GPs and 7 PAs) wished to use the new software long-term. In addition, 3 GPs even stated that they would implement and use the software immediately, if it was linked directly to the EHR software and did not require a second software for bridging the interface. Despite its design to support care management of seniors, the configuration options of eCare*Seniors allowed for applying the software for other patient groups as well. This option was selected by 7 of the 9 practices; 1 practice extended the use to patients aged ≥35 years, 6 practices to all patients. eCare*Seniors was rated positively in comparison with existing patient management strategies of the individual practices (Table 2). The 2 GPs and 2 PAs who did not express a desire to use the new software in the long term said they were already using a well-functioning system which offered the same functionality. All GPs except one were willing to pay a monthly license fee for the software, ranging from 5 to 25 Euros (mean 14.75, SD 5.93).

**Table 2:** Questionnaire answers of general practitioners and practice assistants: Comparison of eCare*Seniors with the current practice-specific patient management approach (N=18). Percentages are reported for valid cases.

Aspects assessed	Strongly disagree, n (%)	Disagree, n (%)	Neither agree nor disagree, n (%)	Agree, n (%)	Strongly agree, n (%)
eCare*Seniors offers a quicker overview of all important patient-centered contents of care	1 (6)	4 (22)	1 (6)	9 (50)	3 (17)
I think that eCare*Seniors gives better reminders about upcoming checkups, vaccinations, and routine examinations	0 (0)	3 (17)	1 (6)	5 (28)	9 (50)
I think that eCare*Seniors better supports individual, guideline-oriented care planning for complex patients	0 (0)	3 (17)	2 (11)	7 (39)	6 (33)
I think that eCare*Seniors better supports time-efficient processes in time-limited everyday practice	0 (0)	2 (11)	5 (28)	3 (17)	8 (44)
I think that the reminder function of eCare*Seniors offers a financial benefit	1 (6)	3 (17)	2 (11)	9 (50)	3 (17)

**Table 3:** Participants’ evaluation of the practicability based on the System Usability Scale (N=18). Percentages are reported for valid cases.

Items of the System Usability Scale	Strongly disagree, n (%)	Disagree, n (%)	Neither agree nor disagree, n (%)	Agree, n (%)	Strongly agree, n (%)
I think that I would like to use this system frequently	0 (0)	4 (22)	3 (17)	6 (33)	5 (28)
I found the system unnecessarily complex	10 (56)	3 (17)	3 (17)	2 (11)	0 (0)
I thought the system was easy to use	0 (0)	1 (56)	2 (11)	7 (39)	8 (44)
I think that I would need the support of a technical person to be able to use this system	7 (39)	8 (44)	1 (6)	0 (0)	2 (11)
I found the various functions in this system were well integrated	0 (0)	3 (17)	4 (22)	10 (56)	1 (6)
I thought there was too much inconsistency in this system	5 (28)	3 (17)	3 (17)	7 (39)	0 (0)
I would imagine that most people would learn to use this system very quickly	0 (0)	1 (6)	0 (0)	10 (56)	7 (39)
I found the system very cumbersome to use	12 (67)	4 (22)	0 (0)	2 (11)	0 (0)
I felt very confident using the system	0 (0)	0 (0)	3 (17)	7 (39)	8 (44)
I needed to learn a lot of things before I could get going with this system	11 (65)	3 (18)	2 (12)	1 (6)	0 (0)

The average SUS score was 75% (SD 14%), varying from 43% to 95%. A total of 14 of the 18 participants (78%) rated the usability as ≥70%, which denotes good usability, although 1 participant (5%) rated the usability as <50%. Stratified for GPs and PAs, the respective SUS score was 78% (SD 16%; range 43%-95%) and 73% (SD 12%; range 53%-93%). Evaluation results on item level are illustrated in Table 3.

## Discussion

### Principal Findings

From the users’ perspective, the new EHR add-on meets its intended purpose; GPs and PAs positively emphasize the intuitive, quick, and comprehensive overview of relevant, patient-centered information provided by eCare*Seniors. Applying the feasibility criteria defined by Bowen et al [30], the new add-on is generally feasible; it meets the users’ demands, is accepted within the target group, has a good usability rating, and no technical problems are encountered during the implementation, as long as practices use a 64-bit operating system. Currently, the biggest challenge is the connectivity between the new add-on and the existing IT infrastructure. In perspective, this will be facilitated by standardized and open interfaces to EHR solutions, which are legally consolidated in Germany since 2017 [36].

In the standardized and validated SUS scale, users rated the usability of eCare*Seniors as 75%, which denotes good usability [32]. As no similar IT approaches were studied in German general practices, the usability cannot be compared with that of other IT solutions. In the literature, only few studies report on user-oriented usability evaluations of newly developed electronic tools supporting patient-centered care management. To a minor extent, comparable approaches are described in 4 studies from the United States and Canada. Furthermore, 3 of these studies assessed the usability of newly developed, EHR-integrated CDSS supporting the standardized, guideline-oriented therapy of patients with chronic pain [37,38] or arterial fibrillation [39] in samples of 4 to 12 GPs and PAs in real primary care scenarios; other than eCare*Seniors these systems only support disease-specific patient management rather than comprehensive, patient-centered management [37-39]. Similar to eCare*Seniors, an approach from the United States aims at priority-oriented restructuring of patient-centered information within the EHR to provide a quick overview of patient’s care needs. On the basis of a previous study on information needs, 4 options for information presentation were developed and tested by 16 physicians using fictive patient cases. However, to our knowledge, this concept was only realized as prototype and has not been transferred to EHR solutions thus far [40]. Despite the limited comparability, these studies report similar usability ratings based on the SUS scores or comparable items [37-40].

Although eCare*Seniors predominantly aims to support health care management of multimorbid seniors, most practices wanted to use it for all their patients. This shows that the demand is not limited to complex patients only, which might be explained by the fact that the current EHR solutions were described as inconvenient, requiring high interaction, and lacking reminders relevant for care [5]. Interestingly, this result contradicts a study by Sittig et al indicating that physicians prefer IT solutions supporting the management of elderly patients with multiple conditions or polypharmacy [15]. Nevertheless, it has to be considered that our approach aimed to overcome deficits of German EHR systems. This also makes it difficult to compare our approach with other studies on CDSS as most studies evaluated the effect of newly developed, often imposed systems instead of further developing existing IT solutions with regard to deficits and users’ needs [10-14].

### Limitations

The key strength of the development and feasibility testing of eCare*Seniors is the user-oriented, bottom-up approach, which facilitated adequate consideration and realization of the users’ demands. In addition, the mixed methods design involved qualitative and quantitative elements allowing for a more detailed assessment of the users’ opinions than a strict quantitative approach. For feasibility testing, the study sample included 18 GPs/PAs and 4 patient cases per participant. Although this is within the range of sample sizes recommended for usability studies [33], the sample analyzed is too small to generate generalizable and reproducible results. Another aspect which limits the results’ generalizability and reproducibility is the fact that the SUS provides self-reported, subjective measures of usability and the study lacks objective usability measures. In addition, the results might be limited because of a selection and response bias. First, it cannot be excluded that the participating practices had a greater interest in new health IT or had a higher affinity for technology than those who refused to participate. Second, the responses of the participants might be positively influenced by the presence of a member of the research team. As GPs and PAs expressed their criticism ad hoc, this bias is considered negligible.

### Conclusions

This feasibility study shows that the newly developed EHR add-on is well accepted and usable; however, the technical integration into present IT infrastructures of general practices could be facilitated. To determine whether the utilization of the software positively influences practice organization, patient care, and patients’ health outcomes, randomized controlled interventional studies are needed.

## Abbreviations

CDSS: clinical decision support system
EHR: electronic health record
GP: general practitioner
IT: information technology
PA: practice assistant
SUS: System Usability Scale
